# Multi-Scale Spatial–Temporal Graph Model for Unsupervised Anomaly Detection in the Wheat Flour Transportation Process

**DOI:** 10.3390/foods15162934

**Published:** 2026-08-21

**Authors:** Wanbao Sheng, Huawei Jiang, Wenqiang Pi, Zhen Yang, Like Zhao

**Affiliations:** 1 College of Information Science and Engineering, Henan University of Technology, Zhengzhou 450001, China; shengwb@stu.haut.edu.cn (W.S.);; 2 Henan Zhongyu Port and Shipping Construction Engineering Co., Ltd., Zhengzhou 450001, China

**Keywords:** anomaly detection in wheat flour transportation, unsupervised learning, graph neural networks, variational autoencoder, Kolmogorov–Arnold networks

## Abstract

Wheat flour transportation involves extended transit periods, which present considerable challenges for safety risk oversight. Therefore, it is essential to develop an efficient anomaly detection method to support risk assessment during this stage. However, existing anomaly detection methods often neglect the coupling effects across different time scales and the spatial clustering of hazard factors. To address this limitation, we propose a multi-scale spatial–temporal graph model-based unsupervised anomaly detection framework (MSTUAD), which can simultaneously capture the spatial–temporal correlations between importance and hazard factors across multiple time scales. Specifically, feature maps are first constructed for each hazard factor within a given time window to represent coupling effects. Secondly, a multi-scale spatial–temporal graph model is designed to extract spatial–temporal characteristics from these feature maps. Finally, a reconstruction model based on a variational autoencoder learns latent representations of spatial–temporal features of hazard factors, thereby capturing the intrinsic characteristics of normal wheat flour. Experimental validation on the wheat flour transportation hazard factor dataset and three public industrial datasets demonstrates that MSTUAD significantly outperforms state-of-the-art anomaly detection methods, achieving an average F1-score greater than 84.55%. This approach provides valuable decision support and technical guidance for relevant regulatory authorities.

## 1. Introduction

Food safety is a critical global issue that is closely linked to human health. As food supply chains become increasingly complex, international food safety incidents continue to occur [1,2,3]. For example, in April 2022, Salmonella contamination was detected in Kinder chocolate products during cross-border distribution in Belgium, prompting a large-scale recall and regulatory investigation across 11 countries with more than 150 cases of foodborne illness [4]. In the same year, inadequate oversight of berry transportation in several European countries resulted in contamination with norovirus and hepatitis A virus [5]. Meanwhile, with the advancement of industrialization and urbanization in China, heavy metals and cyanotoxins in irrigation water can enter the food chain through transport processes, posing significant risks to human health [6]. Furthermore, the recent “3.15” Gala in China revealed that several food companies were excessively adding phosphate water-retaining agents during seafood transportation to preserve product appearance and weight. Long-term consumption of such products may lead to calcium–phosphorus imbalance and cardiovascular disease. As a primary dietary staple, the wheat flour supply chain encompasses production, processing, storage, transportation, and consumption. The transportation stage is particularly vulnerable to factors such as fluctuations in temperature and humidity, as well as packaging damage. These conditions may induce starch denaturation, promote microbial proliferation, and accelerate lipid oxidation, thereby generating both chemical and biological hazards. These hazards may interact through synergistic or antagonistic mechanisms, creating coupled risks that present significant food safety concerns. Therefore, conducting a risk assessment of the wheat flour transportation stage holds substantial practical and applied value [7].

Currently, many scholars investigate interactions among hazard factors using decision-analysis methods, such as the analytic hierarchy process [8] and gray correlation analysis [9], in order to obtain more accurate food-safety risk assessment results. However, these methods rely heavily on expert subjective judgment, which may lead to limited stability and generalization performance when applied to diverse food detection data. In contrast, artificial neural networks update parameters through gradient-descent optimization without requiring expert knowledge and have been widely applied in food computing [10]. Therefore, researchers have integrated decision-analysis techniques with artificial neural networks to enhance the assessment of food safety risks [11,12]. However, actual food safety risks exhibit complex temporal dynamics characteristics influenced by factors such as climatic conditions and storage practices. The above methods generally ignore the temporal dimension of food hazard factors and cannot effectively learn long-term dependencies. As a result, there is a need to develop deep-learning models to identify complex dependencies among hazard factors. Long Short-Term Memory (LSTM) networks exhibit strong capabilities for modeling correlations within long-sequence data and have been widely applied in food-safety risk assessment [13].

Although LSTMs can effectively capture complex relationships among hazard factors when predicting food safety risks, they require preprocessing of hazard factor detection data to generate target outputs for supervised learning. In contrast, anomaly detection uses a data-driven approach to identify samples that differ significantly from the majority. It does not rely on pre-labeled data but instead detects risks by uncovering latent patterns and outliers within the dataset. Existing research has demonstrated the substantial applicability of anomaly detection in food safety monitoring [7,14]. However, current anomaly detection methods neglect the spatial correlations among hazard factors during food transportation. To overcome this limitation, researchers have proposed a new approach for modeling complex spatial relationships. This approach not only constructs graph structures based on node embedding similarity metrics but also employs graph neural networks to learn the spatial relationships among hazard factors and has achieved significant success in the field of food safety risk assessment [15].

However, existing anomaly detection methods focus only on the single-time or spatial structural characteristics of food hazards, neglecting the modeling of dynamic characteristics across different time scales, and are thus unable to effectively integrate short-term and long-term safety risks during transportation. To this end, we propose an unsupervised anomaly detection framework for wheat flour based on a multi-scale spatial–temporal graph model. Specifically, we first employ sliding window techniques to decompose hazard factor data during wheat flour transportation into multiple consecutive time windows, constructing feature maps for each window. Secondly, we design a multi-scale spatial–temporal graph model that integrates multi-scale convolutional attention with spatial–temporal feature extraction. Finally, we learn latent representations of spatial–temporal features in hazard factors through reconstruction learning based on a variational autoencoder to capture characteristics of normal wheat flour. Additionally, we employ end-to-end optimization of the proposed MSTUAD framework using a joint loss function. Its main contributions are as follows:

1. This paper designs a coupled graph structure learning approach that quantifies the interrelationships among hazard factors. This method effectively captures synergistic interactions and antagonistic effects among hazard factors in wheat flour.
2. This paper proposes a novel multi-scale spatial–temporal graph model that employs a multi-scale convolutional attention to learn correlations across multiple temporal scales. By incorporating a spatial–temporal feature extraction module, the model captures the spatial–temporal characteristics of wheat flour hazard factors.
3. This paper improves a reconstruction learning approach based on a variational autoencoder. It employs a Kolmogorov–Arnold network as both the inference and generative networks, thereby enabling more flexible modeling of the latent feature distribution of normal wheat flour through learnable activation functions.

## 2. Materials and Methods

### 2.1. Problem Statement

Given the wheat flour transportation hazard factor dataset $X=\left\{x_{1},x_{2},\ldots ,x_{n}\right\}\in {\mathbb{R}}^{n\times t}$ as training input, the goal of unsupervised anomaly detection is to identify whether previously unseen observations $x_{t}(t>n)$ are anomalous. For anomaly detection in wheat flour transportation, historical values help characterize the current time point. Therefore, we first defined a time window $w_{t}=\left\{x_{t-\tau +1},\ldots ,x_{t-1},x_{t}\right\}$ of length τ at time point *t*, transforming the wheat flour transportation hazard factor dataset into $Y=\left\{w_{1},w_{2},\ldots ,w_{n}\right\}$ as the training input for the model. Secondly, we calculated the anomaly scores for time window $w_{t}(t>n)$. Finally, the anomaly scores were compared against a threshold to determine the binary label *y*, where 1 denotes an anomaly, and 0 denotes a normal observation.

### 2.2. Data Sources and Construction

The experimental data used in this paper were sourced from routine sampling inspections conducted by the Chinese Administration for Market Regulation of China [16], comprising 4865 wheat flour transportation detection records collected from April 2010 to July 2023, with a sampling interval of one day. Since each detection record includes a detection date, we sorted the records chronologically and constructed a hazard factor time series for wheat flour transportation to characterize the dynamic evolution of the transportation process. Meanwhile, to consider both the potential increase in hazard levels caused by improper temperature and humidity control or inadequate packaging integrity and the risks of toxicity and carcinogenicity associated with bioaccumulation and biological activity, we selected heavy metals, mycotoxins, and other contaminants as indicators for evaluating wheat flour transportation risks. These hazard indicators primarily include mercury (Hg), arsenic (As), lead (Pb), cadmium (Cd), chromium (Cr), zearalenone (ZEN), deoxynivalenol (DON), ochratoxin A (OTA), aflatoxin B1 (AFB1), and benzo[a]pyrene (BaP). Furthermore, this paper established anomaly labels according to the maximum allowable limits specified in the food safety standard for maximum contaminant levels (GB 2762-2025) [17] and the food safety standard for maximum mycotoxin levels (GB 2761-2017) [18], which were used for model validation and performance evaluation. The corresponding national food safety standards for each hazard factor are summarized in Table 1.

### 2.3. Overview

**Figure 1:**
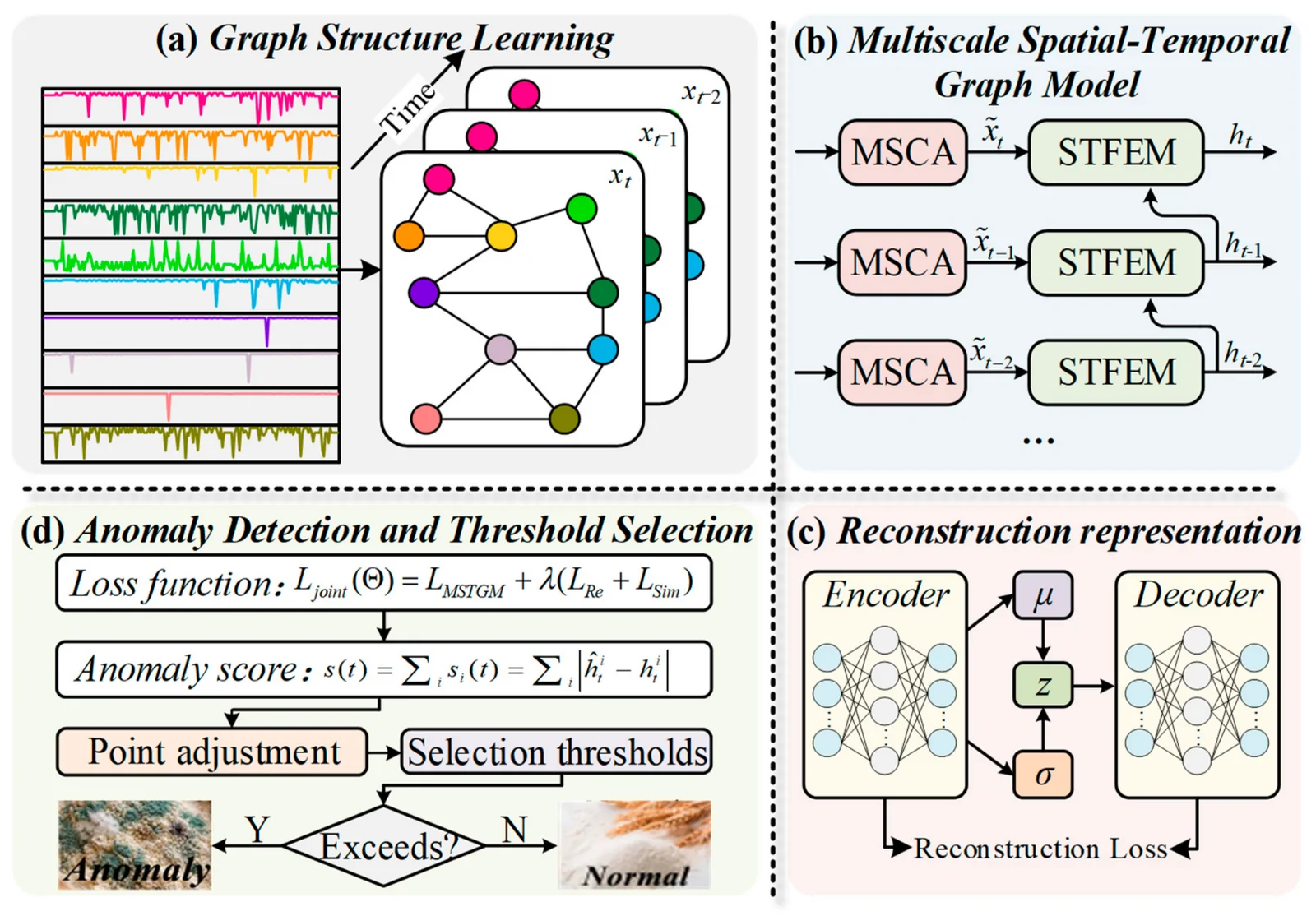
The overview of the proposed MSTUAD. (**a**) Graph structure learning, which converts the sequence of multiple hazard factors in wheat flour transportation into graph structure data using maximum mutual information. (**b**) Multiscale spatial-temporal graph model, which identifies the spatial-temporal characteristics of hazard factors in wheat flour transportation through multi-scale convolutional attention and spatial-temporal feature extraction modules. (**c**) Reconstruction representation, which uses KAN-based Encoder and Decoder to reconstruct spatial-temporal features. (**d**) Anomaly detection and threshold selection, which employs the anomaly score and point adjustment strategy to determine thresholds for detecting anomalies.

In this section, we describe the proposed MSTUAD, which comprises four components: graph structure learning, multi-scale spatial–temporal graph model, reconstruction representation, and anomaly detection with threshold selection, as illustrated in Figure 1. Specifically, graph structure learning uses coupling coefficients to quantify interactions among hazard factors and to construct the adjacency matrix of the representation graph. The multi-scale spatial–temporal graph model applies multi-scale convolutional attention to fuse feature information across different temporal scales and extracts global spatial–temporal information through the spatial–temporal feature extraction module. The reconstruction representation adopts an encoder–decoder architecture based on KAN to reconstruct the spatial–temporal features of hazard factors. Anomaly detection and threshold selection identify abnormal states by computing anomaly scores and determining threshold values using a point-adjusted strategy.

### 2.4. Graph Structure Learning

During transportation, coupled interactions among multiple hazard factors exert significant influence on the overall safety status of wheat flour. But these coupled interactions exhibit nonlinear characteristics, which make them difficult to characterize effectively using traditional statistical methods. Therefore, to present these coupled interactions, this paper transforms the wheat flour hazard factor detection data within each time window into a graph $G_{t}=\left\{V,E\right\}$ consisting of nodes and edges, which is then used as input for a multi-scale spatial–temporal graph model. Here, node $v_{i}\in V$ represents hazard factors, and each hazard factor is connected by edges to its *k* most relevant hazard factors. Specifically, we first use the detection values of each hazard factor within the current time window as features, denoted as $f_{t}^{i}=\left\{x_{t-\tau +1}^{i},\ldots ,x_{t-1}^{i},x_{t}^{i}\right\}$. Secondly, to ensure balanced coverage of all coupling relationships, we quantify the coupling interactions (including synergistic and antagonistic effects) among the *m* hazard factors based on maximal information [19] and compute the coupling coefficient matrix $C\in {\mathbb{R}}^{m\times m}$:

(1) $$C(f_{t}^{i},f_{t}^{j})=\underset{a*b<B}{\max}\frac{\iint p(f_{t}^{i},f_{t}^{j})\log\left(\frac{p(f_{t}^{i},f_{t}^{j})}{p(f_{t}^{i})p(f_{t}^{j})}\right)df_{t}^{i}df_{t}^{j}}{\log_{2}\min(a,b)},\;i,\;j\in \left\{1,\;2,\;\ldots ,\;M\right\}$$

where *a* and *b* denote the number of grid points in the *x* and *y* directions after two-dimensional grid partitioning, respectively; *B* represents a constant, set to 0.6 in this paper; $p(f_{t}^{i},f_{t}^{j})$ is the joint probability distribution function of $f_{t}^{i}$ and $f_{t}^{j}$; and $p(f_{t}^{i})$ and $p(f_{t}^{j})$ are the marginal probability distribution functions of $f_{t}^{i}$ and $f_{t}^{j}$, respectively.

Finally, high coupling coefficients indicate the presence of coupling effects among hazard factors within the current time window. Thus, this paper employs the k-nearest neighbors (k-NN) approach to identify, for each hazard factor, the *k* hazard factors with the highest coupling coefficients, which together constitute its set of coupling effects. An adjacency matrix is subsequently constructed to implicitly represent the coupling effects among hazard factors:

(2) $$A_{t}(f_{t}^{i},f_{t}^{j})=\left\{\begin{array}{l} 1,j\in topk(\rho (f_{t}^{i},f_{t}^{k}):k\in \left\{1,\;2,\;\ldots ,\;M\right\})\\ 0,otherwise \end{array}\right.$$

### 2.5. Multi-Scale Spatial–Temporal Graph Model

**Figure 2:**
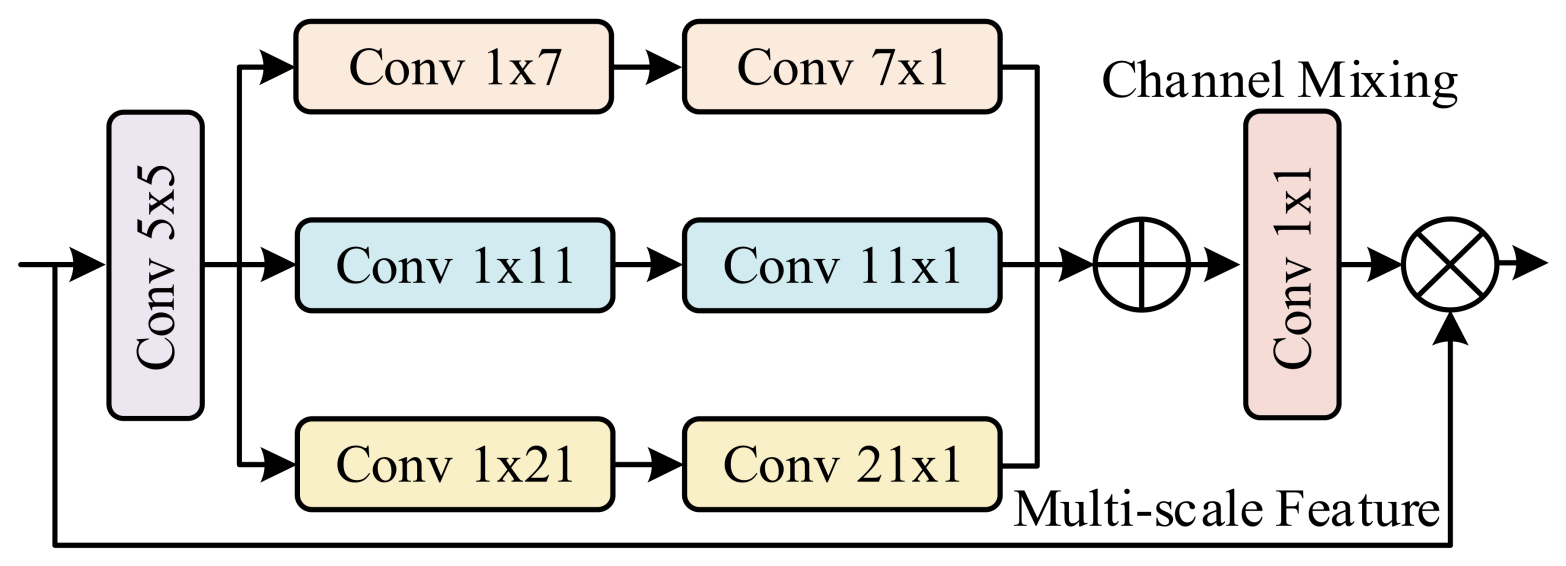
The structure of multi-scale convolutional attention.

Graph structure learning only captures positional information within the current time window *w_t_*, failing to integrate long-term and short-term dynamic trends across multiple windows. To address this limitation, we design a multi-scale spatial–temporal graph model (MSTGM). Specifically, a multi-scale convolutional attention (MSCA) [20] is employed to capture hazard factor features in wheat flour across multi-scale time windows. It consists of three components: deep convolutions that aggregate local information, multi-branch deep strip convolutions that capture multi-scale contextual information, and 1 × 1 convolutions that model relationships across channels, whose mathematical equation is given by

(3) $${\tilde{x}}_{t}=x_{t}⊗Conv_{1\times 1}\left(\sum_{i=0}^{3}Scale_{i}(DWConv(x_{t}))\right)$$

where *x_t_* denotes the *t*th feature within the time window; ⊗ represents element-wise matrix multiplication; *DWConv* indicates depthwise convolution; and $Scale_{i},i\in \{0,1,2\}$ signifies the *i*th branch, whose structure is illustrated in Figure 2.

Meanwhile, to comprehensively consider the spatial–temporal characteristics of hazard factors during wheat flour transportation, this paper designs a spatial–temporal feature extraction module (STFEM) that integrates spatial graph convolution with sequence modeling. Specifically, graph convolution is applied to each graph *G_t_* to aggregate hazard factor information from neighboring regions while maintaining weight-sharing properties, capturing potential coupling relationships among hazard factors:

(4) $$GCN(H_{t}^{l})={\tilde{\Lambda }}^{-\frac{1}{2}}{\tilde{A}}_{t}{\tilde{\Lambda }}^{-\frac{1}{2}}H_{t}^{l}W^{l}$$

where *W^l^* denotes the trainable parameter matrix of layer *l*, ${\tilde{A}}_{t}$ denotes the sum of the adjacency matrix and the identity matrix, $\tilde{\Lambda }$ denotes the degree matrix corresponding to ${\tilde{A}}_{t}$, and $H_{t}^{l}\in {\mathbb{R}}^{n\times d}$ denotes the output of layer *l* for time window $H_{t}^{l}\in {\mathbb{R}}^{n\times d}$.

However, to obtain a larger receptive field, it is often necessary to stack multiple GCN layers, which may result in indistinguishable node features after convergence and vanishing gradients during backpropagation [21]. In practice, most state-of-the-art GCN models do not exceed four layers. Therefore, this paper proposes a residual graph convolutional network (RGCN), which employs skip connections with activation functions across three cascaded GCN layers, described as follows:

(5) $$RGCN=GCN\left[ReLU\left(GCN\left(ReLU\left(GCN\left({\tilde{x}}_{t}\right)\right)\right)\right)\right]⊕{\tilde{x}}_{t}$$

where *GCN*(.) denotes graph convolution, *ReLU*(.) denotes the activation function, and ⊕ denotes element-wise summation.

In addition, previous studies have demonstrated that LSTM can capture the temporal dependencies of hazard factors in wheat flour [22]. Therefore, to jointly capture spatial–temporal dependencies, the proposed RGCN is incorporated into the LSTM through matrix multiplication, enabling the extraction of broader spatial features. Specifically, each LSTM unit contains two internal states, *c_t_* and *h_t_*. The cell state *c_t_* is regulated by three gates: the forget gate ft, the input gate it, and the output gate *o_t_*. Since deep neural networks are prone to vanishing or exploding gradients during training, an exponential gating function is introduced as the activation function in both the input and forget gates. In addition, a normalized state is incorporated to stabilize gradient propagation, thereby reducing the risk of gradient vanishing or explosion and improving the stability of model training:

(6) $$i_{t}=(RGCN\circ exp)(W_{f}[h_{t-1};{\tilde{x}}_{t}]+b_{f})$$

(7) $$i_{t}=(RGCN\circ exp)(W_{i}[h_{t-1};{\tilde{x}}_{t}]+b_{i})$$

(8) $$o_{t}=(RGCN\circ \sigma )(W_{o}[h_{t-1};{\tilde{x}}_{t}]+b_{o})$$

(9) $$c_{t}=f_{t}⊗c_{t-1}+i_{t}⊗(RGCN\circ \tanh)(W_{c}[h_{t-1};{\tilde{x}}_{t}]+b_{c})$$

(10) $$n_{t}=f_{t}⊗n_{t-1}+i_{t}$$

(11) $${\tilde{h}}_{t}=c_{t}/n_{t}$$

(12) $$h_{t}=o_{t}⊗{\tilde{h}}_{t}$$

where [;] denotes a series operation; *h_t_*_−1_ represents the previous hidden state; ${\tilde{x}}_{t}$ denotes the current input; σ denotes the sigmoid activation function; *exp* denotes the exponential gate activation function; and ∘ and ⊗ denote functional composition and element-wise multiplication, respectively. *W_f_*, *W_i_*, *W_o_*, *W_c_* and *b_f_*, *b_i_*, *b_o_*, *b_c_* are learnable parameters; *n_t_* denotes the normalized state at the current time step; *n_t_*_-1_ denotes the normalized state at the previous time step; and ${\tilde{h}}_{t}$ denotes the normalized hidden state at the current time step.

**Figure 3:**
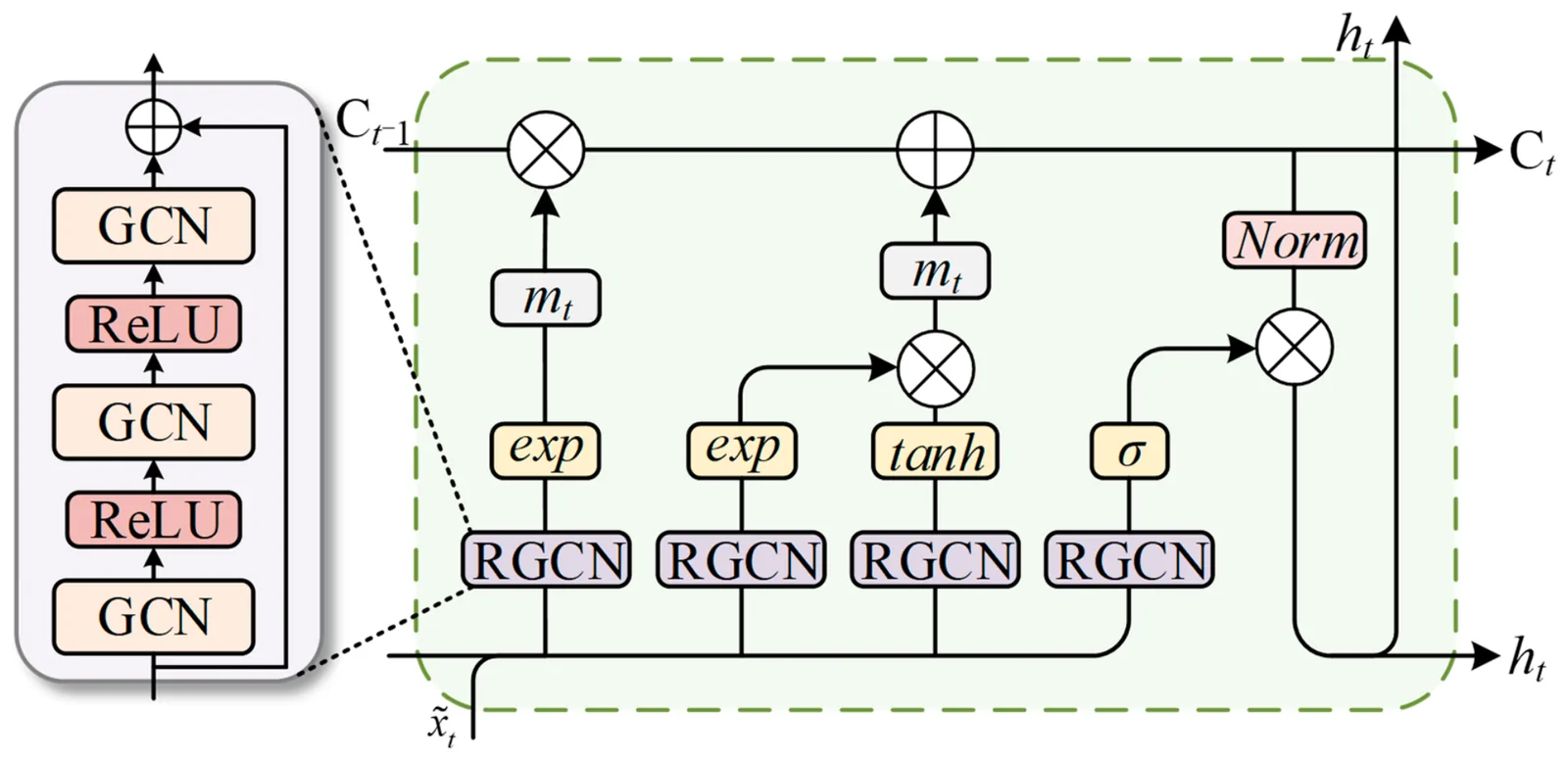
The structure of STFEM.

During the computation of the input and forget gates, the exponential gating activation function may exhibit numerical overflow. To this end, a steady-state parameter, *m_t_*, is designed to regulate the numerical range of the exponential gating activation function, enhancing model stability and improving the accuracy of spatial–temporal feature extraction for hazard factors:

(13) $$m_{t}=\max\left(\log\left(f_{t}\right)+m_{t-1},\log(i_{t})\right)$$

(14) $${i_{t}}^{\prime }=\exp\left(\log\left(i_{t}\right)-m_{t}\right)$$

(15) $${f_{t}}^{\prime }=\exp\left(\log\left(f_{t}\right)+m_{t-1}-m_{t}\right)$$

where *m_t_* denotes the stable state at the current time step, *m_t_*_−1_ denotes the stable state at the previous time step, and *i_t_* and *f_t_* denote the values of the output gate and forget gate, respectively. The overall architecture of the STFEM is illustrated in Figure 3. This approach not only handles extreme values more effectively, thereby reducing the impact of anomalous data on model performance, but also substantially enhances model stability. Moreover, it achieves performance comparable to that of more complex models while maintaining low computational complexity.

### 2.6. Reconstruction Representation

**Figure 4:**
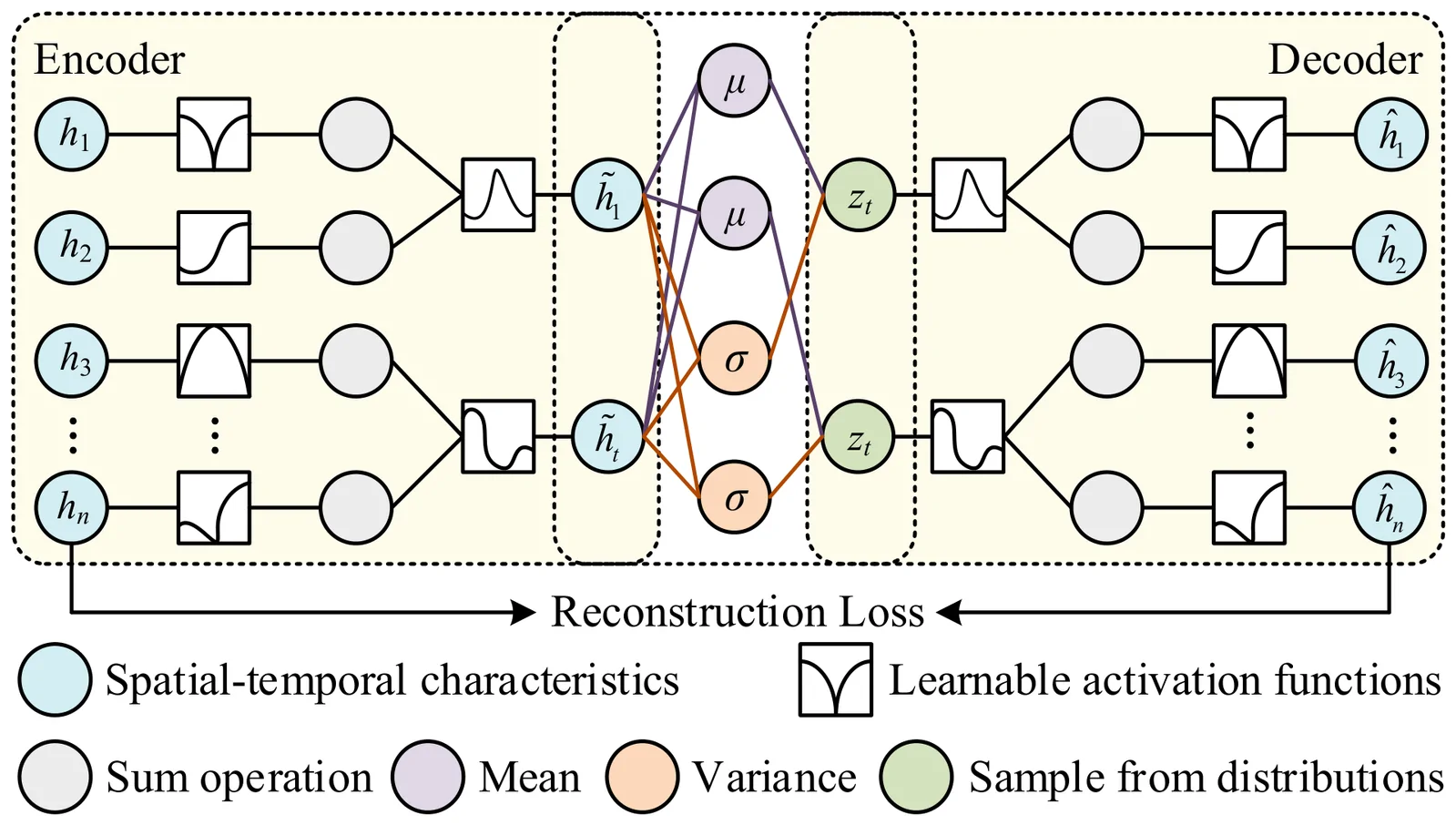
The structure of an improved variational autocoder.

Although a multi-scale spatial–temporal graph model can capture dependencies across temporal and spatial dimensions, accurately distinguishing between normal and abnormal wheat flour remains crucial for unsupervised anomaly detection. Variational Autoencoder (VAE) is capable of extracting latent patterns from high-dimensional data and has been widely applied to anomaly detection tasks [23]. Specifically, VAE employs dimensionality reduction to compress the spatial–temporal feature representation *h_t_* of the wheat flour hazard factor into a low-dimensional latent variable *z_t_* and subsequently reconstructs *h_t_* from *z_t_*. Assuming that *z_t_* follows a prior distribution *p_θ_*(*z_t_*), the reconstructed feature *ĥ_t_* can be sampled from the posterior distribution *p_θ_*(*h_t_*|*z_t_*). However, conventional VAEs typically employ a multi-layer perceptron (MLP) as both inference and generative networks, which can lead to poor interpretability and high parameter complexity in wheat flour anomaly detection tasks. Kolmogorov–Arnold networks (KANs) [24] employ learnable activation functions to parameterize weights in spline form, offering high flexibility and enabling complex functions to be simulated with fewer parameters, thereby improving model interpretability. Building on this idea, the traditional VAE is enhanced by employing KAN as both the inference network *q_φ_*(*z_t_*|*h_t_*) and the generative network *p_θ_*(*h_t_*|*z_t_*), where *φ* and *θ* denote the parameters of the inference and generative networks, respectively. The internal architecture of the proposed model is illustrated in Figure 4.

As can be seen from Figure 4, learnable activation functions and spline functions are first applied to *h_t_* to reduce the embedding dimensionality of the spatial–temporal features of the wheat flour hazard factor, ensuring that they approximately follow a normal distribution. The mean and variance are then computed to obtain the latent representation vector *z_t_*:

(16) $$z_{t}(\mu ,\sigma )=\sum_{k=1}^{K}\omega_{k}\phi_{k}(S_{k}(h_{t}))$$

where $\sum_{k=1}^{K}\omega_{k}\phi_{k}(\cdot )$ represents the spline function of the encoder, *S_k_*(.) denotes the learnable activation function of the encoder, *μ* denotes the mean, and *σ* denotes the variance. Secondly, the features are reconstructed using the spline function and the learnable activation function within the decoder:

(17) $${\hat{h}}_{t}=\sum_{k=1}^{K}\omega_{k}^{\prime }\varphi_{k}^{\prime }(S_{k}^{\prime }(z_{t}))$$

where $\sum_{k=1}^{K}\omega_{k}^{\prime }\varphi_{k}^{\prime }(\cdot )$ represents the spline function of the decoder, and $S_{k}^{\prime }(\cdot )$ denotes the learnable activation function of the decoder. Finally, model parameters are optimized by adjusting the spline function and the learnable activation function through the reconstruction loss.

### 2.7. Anomaly Detection and Threshold Selection

#### 2.7.1. Offline Training

To achieve optimal detection performance, a joint loss function is designed for end-to-end training of the MSTUAD framework. Specifically, for unsupervised learning, to optimize the multi-scale spatial–temporal graph model, we employ negative sampling and utilize a binary cross-entropy loss function to maximally enhance similarity among node embeddings in positive samples and minimize similarity among node embeddings in negative samples:

(18) $$L_{MSTGM}=-\sum_{i=1,j=1}\log\sigma \left[Dot(c_{t}^{i},{\left(c_{t}^{j}\right)}^{T})\right]-\sum_{k=1,l=1}\log\sigma \left[Dot(d_{t}^{k},{\left(d_{t}^{l}\right)}^{T})\right]$$

where $c_{t}^{i}$ and $d_{t}^{k}$ represent the sets of positive and negative samples, respectively, for the embedding vector in time window *W_t_*, *T* represents matrix transposition, and *Dot*(.) denotes the inner product.

Secondly, consistent with most VAE training approaches, stochastic gradient variational Bayes (SGVB) [25] is employed to optimize the VAE parameters by maximizing the evidence lower bound (ELBO). For the spatial–temporal feature *h_t_* of the wheat flour hazard factor, the reconstruction loss function *L_Re_* and the similarity metric function *L_Sim_* are respectively defined as follows:

(19) $$L_{Re}=\frac{1}{K}\sum_{i=1}^{K}{\left({\hat{h}}_{t}^{i}-h_{t}^{i}\right)}^{2}$$

(20) $$L_{Sim}=D_{KL}\left[q_{\phi }(z_{t}|h_{t})\left\|p_{\theta }(z_{t})\right.\right]$$

where ${\hat{h}}_{t}^{i}$ and $x_{t}^{i}$ denote the reconstructed value and the actual value of the *i*th variable in time window *w_t_*, respectively, and $D_{KL}\left[q_{\phi }(z_{t}|h_{t})\left\|p_{\theta }(z_{t})\right.\right]$ represents the regularization term for the latent variable *z_t_*, which is obtained by minimizing the Kullback–Leibler divergence between the approximate posterior and the prior distribution of the latent variable.

Finally, we integrate *L_MSTGM_*, *L_Re_*, and *L_Sim_* to jointly train the proposed MSTUAD, measuring the importance of the multi-scale spatial–temporal graph model and the reconstruction representation through the balanced parameter *λ*. The joint training loss function is

(21) $$L_{joint}(\Theta )=L_{MSTGM}+\lambda (L_{Re}+L_{Sim})$$

where Θ denotes all parameters requiring training, including *W^l^*, *W_f_*, *W_i_*, *W_o_*, *W_c_*, *b_f_*, *b_i_*, *b_o_*, *b_c_*, Φ, and *θ*.

#### 2.7.2. Online Detection

After training, the MSTUAD can be used to detect abnormal conditions that deviate from normal patterns in wheat flour. Specifically, this paper calculates a comprehensive anomaly score by aggregating the anomaly scores at each time point within a time window. If the anomaly score of time window *w_t_* exceeds the threshold, the corresponding time window is classified as anomalous. The anomaly score is calculated as follows:

(22) $$s(t)=\sum_{i}s_{i}(t)=\sum_{i}\left|{\hat{h}}_{t}^{i}-h_{t}^{i}\right|$$

At the same time, to determine the optimal threshold, this study sets the search iterations to 700 with a step size of 0.01 and performs threshold optimization based on the F1-score of the validation set. The threshold achieving the highest validation F1-score is selected as the optimal threshold. Additionally, a point-adjustment strategy is applied to the anomaly scores based on the method proposed in reference [26]. Algorithm 1 summarizes the main procedure of the MSTUAD.


**Algorithm 1** Pseudo-code of MSTUAD framework under the guidance of loss function**Input:** Wheat flour multi-hazard factor detection data *X*, parameters *n*, *τ*, *k*.**Output:** The value of loss.1: **for**
*t* = *τ* to *n* **do**2:               *w_t_* = *x* [*t* − *τ* + 1:*t*]3:               *ρ* ← MIC (*w_t_*)4:               *G_t_* ← *k*-NN (*k*, *ρ*)5:               ${\tilde{x}}_{t}$ ← MSCA (*x_t_*)6:               *h_t_*←STRCM $\left({\tilde{x}}_{t},G_{t}\right)$7:               ${\hat{h}}_{t}\leftarrow \;\mathrm{VAE}(h_{t})$8: **end for**9: Calculate *L_joint_* using Equation (22)10: Back propagation and update parameters in MSKTSAD11: Return *L_joint_*


## 3. Experiments and Results

In this section, we first provide a detailed introduction to the experimental details and baseline models. Secondly, we compare our proposed MSTUAD with state-of-the-art baseline methods, demonstrating its effectiveness in wheat flour anomaly detection. Subsequently, we focus on ablation experiments to validate the impact of each component within the model. Finally, we discuss the model’s broad applicability and parameter sensitivity.

### 3.1. Experimental Details

#### 3.1.1. Data Preprocessing

Due to differences in the physical interpretations and measurement units of hazard factors during wheat flour transportation, Min-Max normalization is applied to standardize the training set, while the normalization parameters (mean and variance) are applied to the test set to prevent data leakage:

(23) $$x_{i}^{*}=\frac{x_{i}-x_{min}}{x_{max}-x_{min}}$$

where $x_{i}=\left\{x_{1},x_{2},\ldots ,x_{T}\right\}$ denotes the wheat flour transportation hazard factor dataset, $x_{i}^{*}$ denotes the normalized data, *x_max_* denotes the maximum value of the dataset, and *x_min_* denotes the minimum value of the dataset.

#### 3.1.2. Baseline Model

The proposed MSTUAD is compared with state-of-the-art baseline models, including machine learning-based anomaly detection methods and widely used deep-learning-based multivariate anomaly detection approaches. Detailed descriptions of the baseline models are provided in Table 2.

#### 3.1.3. Setting of Hyperparameters

The MSTUAD framework is implemented using Python 3.8 and PyTorch 1.11. All experiments were conducted on a workstation equipped with an Intel^®^ Xeon^®^ Silver 4210R CPU and an NVIDIA GeForce RTX 3090 GPU. In this experiment, the wheat flour transportation hazard factor dataset was divided chronologically into training and test sets at a ratio of 7:3, and 35% of the test set was further allocated as a validation set for determining the optimal threshold. Additionally, to avoid potential information leakage caused by overlapping sliding windows across different subsets, we constructed sliding windows independently within the training and test sets after data partitioning, thereby providing a more reliable evaluation of the model’s generalization capability.

For MSTUAD, the number of graph neural network layers l is set to 3, the variable embedding dimension to 5, the time window *τ* to 20, the number of nearest neighbors *k* to 6, the balanced parameter *λ* to 1, the latent representation dimension *z* to 100, and the batch size to 256. At the same time, we evaluated the baseline model using the same time window, normalization method, and threshold strategy. All experimental results are reported as the average of five independent runs with different random seeds. The source code is publicly available at https://github.com/ShengWB/MSTUAD, accessed on 2 July 2026.

#### 3.1.4. Evaluation Metrics

We evaluate the performance of our proposed model and baseline models using *Precision* (*Pre*), *Recall* (*Rec*), *F1-score* (*F1*), and *AUC*, defined as follows:

(24) $$Precision=\frac{TP}{TP+FP}$$

(25) $$Recall=\frac{TP}{TP+FN}$$

(26) $$F1-score=\frac{2\times Precision\times Recall}{Precision+Recall}$$

(27) $$AUC=\int_{0}^{1}Recall(FPR)d(FPR)$$

where *TP* denotes the actual number of anomalies detected, *TN* denotes the actual number of normal instances detected, *FP* denotes the number of anomalies falsely detected, *FN* denotes the number of anomalies undetected, and FPR=FP/FP+TN denotes the false positive rate.

### 3.2. Results and Analysis

To validate the anomaly detection performance of the proposed MSTUAD, comparative experiments were conducted using the wheat flour transportation hazard factor dataset. The results are summarized in Table 3, where the best results are highlighted in bold, and the second-best results are underlined.

As shown in Table 3, MSTUAD achieves substantial improvements in *AUC*, *Pre*, and *F1*. Compared with RNN-based models such as LSTM-NDT, OmniAnomaly, and MSCRED, the *AUC* increases by an average of 32.5%, *Pre* by an average of 38.8%, and *F1* by an average of 18.4%. This is because graph structure learning fully exploits the latent associations among risk factors, enhancing the model’s ability to detect complex anomaly patterns, thereby enabling more accurate detection of anomalies in wheat flour transportation. However, MSTUAD is capable of comprehensively accounting for both local deviations in individual hazard factors and complex interactions among multiple hazard factors. For minor anomalies characterized by small deviations that do not significantly disrupt the learned spatiotemporal correlation patterns, the model tends to regard them as normal fluctuations, resulting in a slight decrease in *Rec*.

Compared with graph neural network-based models, such as GDN, AGCSL, MTAD-GAT, and MUTANT, the *AUC* improved by an average of 17.7%, *Pre* by an average of 24.9%, and *F1* by an average of 11.2%. This improvement results from the multi-scale convolutional attention, which focuses on changes in wheat flour hazard factors across multiple temporal scales, capturing both short-term and long-term temporal dependencies. This enhancement improves the model’s ability to perceive local details, thereby increasing the accuracy of wheat flour anomaly detection.

Compared with encoder-based models, such as TranAD, CAE-M, MEMTO, DTAAD, LFTSAD, and TransDe, the *AUC* improved by an average of 20.1%, *Pre* by an average of 16.6%, *Rec* by an average of 35.6%, and *F1* by an average of 26.1%. This improvement is attributed to the reconstruction learning design, which reduces computational parameters through learnable activation functions and enhances model flexibility, thereby improving MSTUAD’s anomaly detection performance.

### 3.3. Ablation Experiments

**Figure 5:**
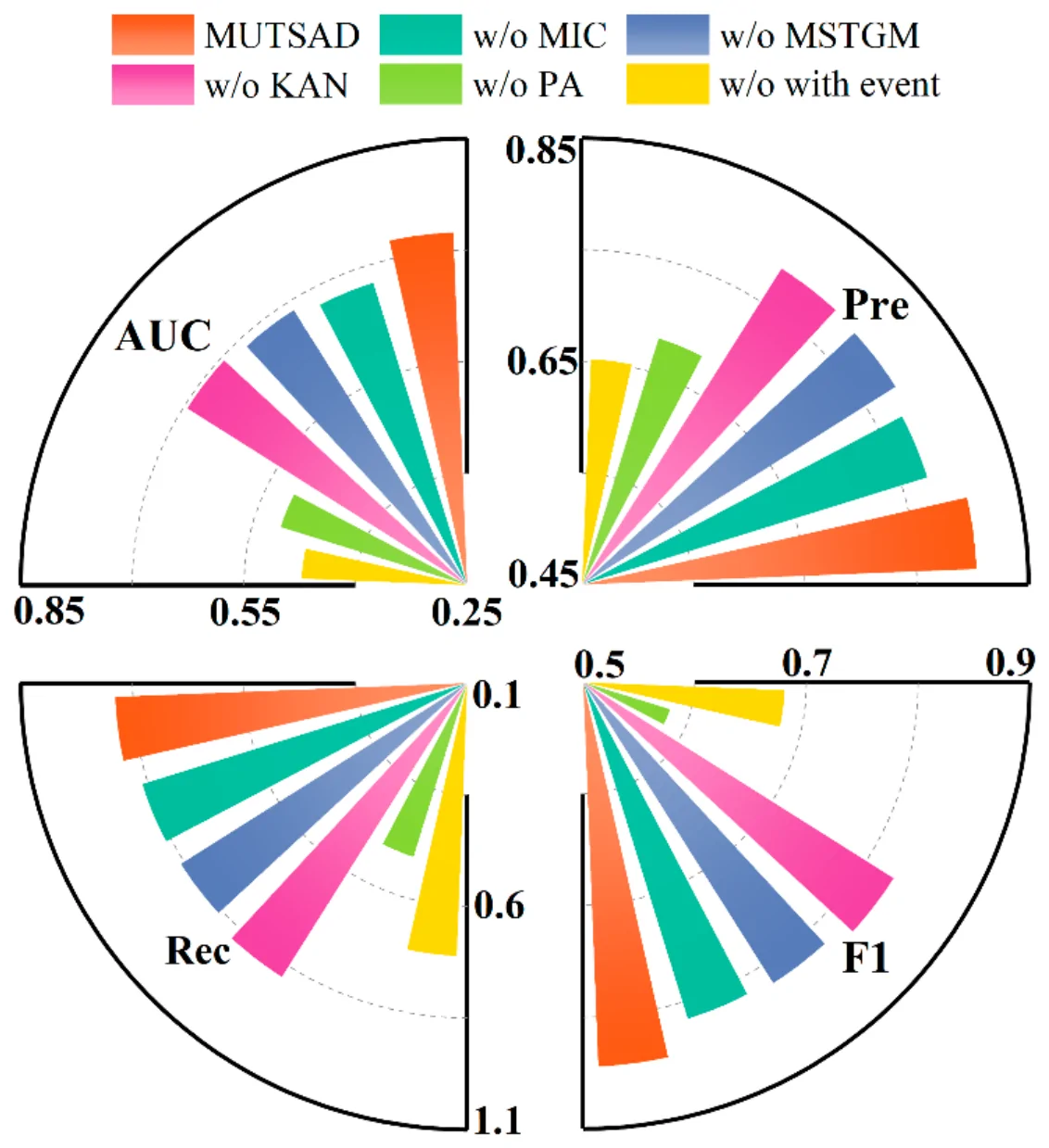
The performance comparison of variants on the wheat flour transportation hazard factor dataset.

To validate the effectiveness and superiority of MIC, MSTGM, and KAN, five ablation variants were meticulously designed for comparative experiments with MSTUAD. Despite partial modifications, all ablation variants retain identical framework structures and parameter settings. The *Pre*, *Rec*, *F1*, and *AUC* of these variants on the wheat flour transportation hazard factor dataset are presented in Figure 5.

(1) **w/o MIC**: This ablation variant removes the maximum mutual information coefficient, using the traditional Pearson correlation coefficient as the standard for measuring the association between hazard factors.
(2) **w/o MSTGM**: This ablation variant removes the multi-scale spatial–temporal graph model and directly uses the time window *w_t_* as input for the reconstruction learning.
(3) **w/o KAN**: This ablation variant removes the KAN layer and employs an MLP layer as both the inference network and the generative network of the VAE.
(4) **w/o PA**: This ablation variant omits the point adjustment strategy and directly uses the computed anomaly scores to calculate the optimal threshold.
(5) **with event**: This ablation variant employs an event-based anomaly detection strategy.

As can be seen from Figure 5, compared **with w/o MIC**, MSTUAD achieved improvements of 6.8%, 3.5%, 3.9%, and 3.7% in *AUC*, *Pre*, *Rec*, and *F1*, respectively. This indicates that the maximum mutual information coefficient effectively encompasses all adjacency relationships, capturing both synergistic and antagonistic interactions among hazard factors. Consequently, MSTUAD demonstrates enhanced adaptability and robustness in graph structure learning, thereby establishing a strong foundation for more accurate and efficient anomaly detection.

Compared with **w/o MSTGM**, MSTUAD exhibits superior performance. Specifically, MSTUAD achieves a 3.3% improvement in *F1*, highlighting that the multi-scale spatial–temporal graph model not only captures dynamic trends and intrinsic correlations across time windows but also uncovers latent dependencies and chronic cumulative effects among wheat flour hazard factors, enhancing the model’s capacity to identify anomaly patterns in complex scenarios.

Compared **with w/o KAN**, although *F1* of MSTUAD improved by only 2%, it leverages the Kolmogorov–Arnold theorem to establish robust nonlinear modeling capabilities at the theoretical level. This unique advantage not only captures spatial–temporal variation patterns among hazard factors but also ensures high interpretability while maintaining accuracy, enhancing the precision of anomaly detection in wheat flour transportation processes.

Compared with **w/o PA** and **with an event**, MSTUAD demonstrates a more substantial performance improvement after incorporating the point adjustment strategy. This indicates that the strategy enables fine-grained correction of point-level detection results under the guidance of event-level cues, thereby preserving the recall advantage offered by event semantics while improving anomaly localization and classification accuracy through point-level refinement, ultimately reducing both false positives and false negatives more effectively.

### 3.4. Parameter Sensitivity

**Figure 6:**
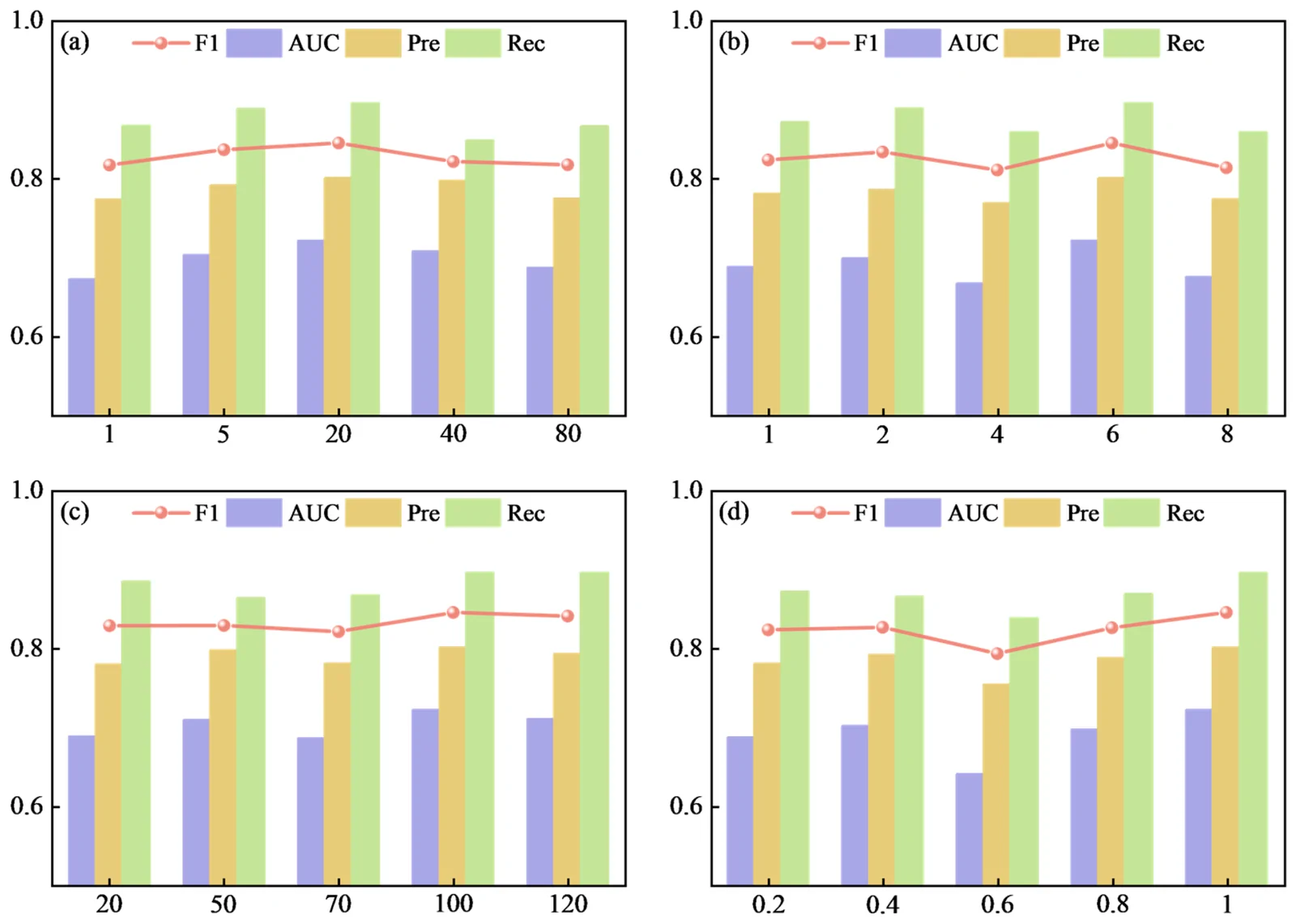
Experimental results of the MSTUAD parameter on the wheat flour transportation hazard factor dataset: (**a**) window length *τ*; (**b**) number of nearest neighbors *k*; (**c**) latent representation layer dimension *z*; (**d**) balanced parameter *λ*.

In practical applications, parameter sensitivity influences the performance of deep-learning models in detecting wheat flour anomalies across various scenarios. Therefore, this section examines the impact of four key parameters in the MSTUAD on anomaly detection performance: time window length *τ*, number of nearest neighbors *k*, latent representation layer dimension *z*, and balanced parameter *λ*. The results are presented in Figure 6.

Figure 6a illustrates the Pre, Rec, F1, and AUC under different time window lengths. It can be observed that as the time window length increases, the performance of MSTUAD initially improves and subsequently declines. When *τ =* 20, MSTUAD achieves optimal performance. This occurs because when the time window is too small, the limited amount of data prevents the model from effectively learning synergistic or antagonistic interaction patterns among hazard factors. However, excessively large time windows introduce overly complex information, which complicates the interrelationships among hazard factors and ultimately degrades wheat flour anomaly detection performance.

The second parameter researched is the number of nearest neighbors k, which determines how many similar hazard factors are selected as neighboring nodes for a given hazard factor. As shown in Figure 6b, the impact of k on the model’s detection performance exhibits an initial increase followed by a subsequent decline. MSTUAD achieves optimal performance when *k* = 6. If *k* is too small, the model may overlook coupling relationships among hazard factors. Conversely, when *k* is excessively large, noise may weaken the true correlations among hazard factors.

Figure 6c indicates that, as the dimension of the latent representation layer increases, the performance of MSTUAD gradually declines and then exhibits a slight improvement. When *z* = 100, MSTUAD achieves optimal performance. The primary reason is that a smaller latent representation dimension leads to the loss of critical information, resulting in larger reconstruction errors for normal wheat flour samples. Conversely, an excessively large latent dimension fails to capture essential features, preventing the model from effectively distinguishing abnormal wheat flour from normal samples.

Figure 6d illustrates the effect of the balanced parameter *λ* on anomaly detection performance. It can be observed that the influence of the balanced parameter *λ* on the model exhibits a trend similar to that of the number of nearest neighbors *k*. When *λ* = 1, MSTUAD achieves optimal performance. This is because the balanced parameter *λ* regulates the relative importance of the multi-scale spatial–temporal graph model and the reconstruction representation during training. An appropriate *λ* not only enables the model to capture latent temporal and spatial structural features among hazard factors but also facilitates accurate reconstruction of their spatial–temporal characteristics, enhancing the detection capability for abnormal wheat flour.

### 3.5. Extensive Experiments

To further validate the general applicability of the proposed MSTUAD, we conducted comparative experiments on three publicly available real-world datasets: the Mars Science Laboratory Rover (MSL) [28], the Soil Moisture Active Passive satellite (SMAP) [28], and the Safe Water Treatment [41]. Table 4 summarizes the detailed statistics of the three datasets. In the experiment, we used the training set shown in Table 4 to train the anomaly detection models and the test set to evaluate the performance of all models. The specific parameter settings and details of the evaluation process also follow Section 3.1.3.

Table 5 presents the performance results of all methods across the three public datasets, with the best results highlighted in bold and the second-best results underlined. It is evident that, across the three public datasets, MSTUAD consistently outperforms all baseline methods in terms of *F1*. Specifically, compared with the best-performing baseline model across all public datasets, MSTUAD achieves an average *F1* improvement of 0.68%. This result validates the stability of MSTUAD across different datasets and highlights its broad applicability in practical scenarios.

Compared with traditional anomaly detection methods, MSTUAD demonstrates consistently superior performance across the three public datasets. When compared with prediction-based anomaly detection methods, MSTUAD achieves average *F1* improvements of 61.5%, 73%, and 112.6%, respectively, across the three public datasets. Compared with reconstruction-based anomaly detection methods, MSTUAD achieves average *F1* improvements of 26.9%, 30.4%, and 18.1% across the three public datasets. Compared with contrastive learning-based anomaly detection methods, MSTUAD achieves an average *F1* improvement of 0.95% across the three public datasets.

Furthermore, compared with OmniAnomaly, MTAD-GAT, DTAAD, and LFTSAD, MSTUAD achieves average *F1* improvements of 20.3% and 12.2% on small-scale datasets (i.e., MSL and SWaT), respectively, as well as an average *F1* improvement of 34.2% on large-scale datasets (i.e., SMAP). This improvement can be attributed to MSTUAD’s ability to effectively capture interdependencies among multiple variables, thereby enhancing detection performance on large-scale datasets.

### 3.6. Complexity Analysis

Model complexity serves as an indicator of the model’s computational resource requirements. Table 6 summarizes the parameter counts and training times of MSTUAD in comparison with other methods. Notably, to more intuitively illustrate the balance between complexity and detection accuracy, the F1 on the wheat flour transportation hazard factor dataset was selected as the evaluation metric for these approaches.

As shown in Table 6, the GDN and DTAAD methods exhibit relatively low parameter counts. This is because these approaches employ graph neural networks and shallow temporal convolutional networks, respectively, to capture inter-variable relationships or temporal dependencies, thereby reducing model complexity. Although MSTUAD is constructed based on graph neural networks and shallow recurrent neural networks, it exhibits a comparatively larger number of parameters and the longest training time. This is because MSTUAD incorporates multi-scale convolutional attention and residual graph neural networks, which stack numerous convolutional operations and consequently increase model complexity. Nevertheless, compared with GDN and DTAAD, MSTUAD achieves a superior *F1*. Furthermore, methods such as ACGSL, CAE-M, and LFTSAD exhibit lower model complexity due to their encoder–decoder architectures. However, their anomaly detection accuracy remains inferior to the proposed MSTUAD.

### 3.7. Case Study

**Figure 7:**
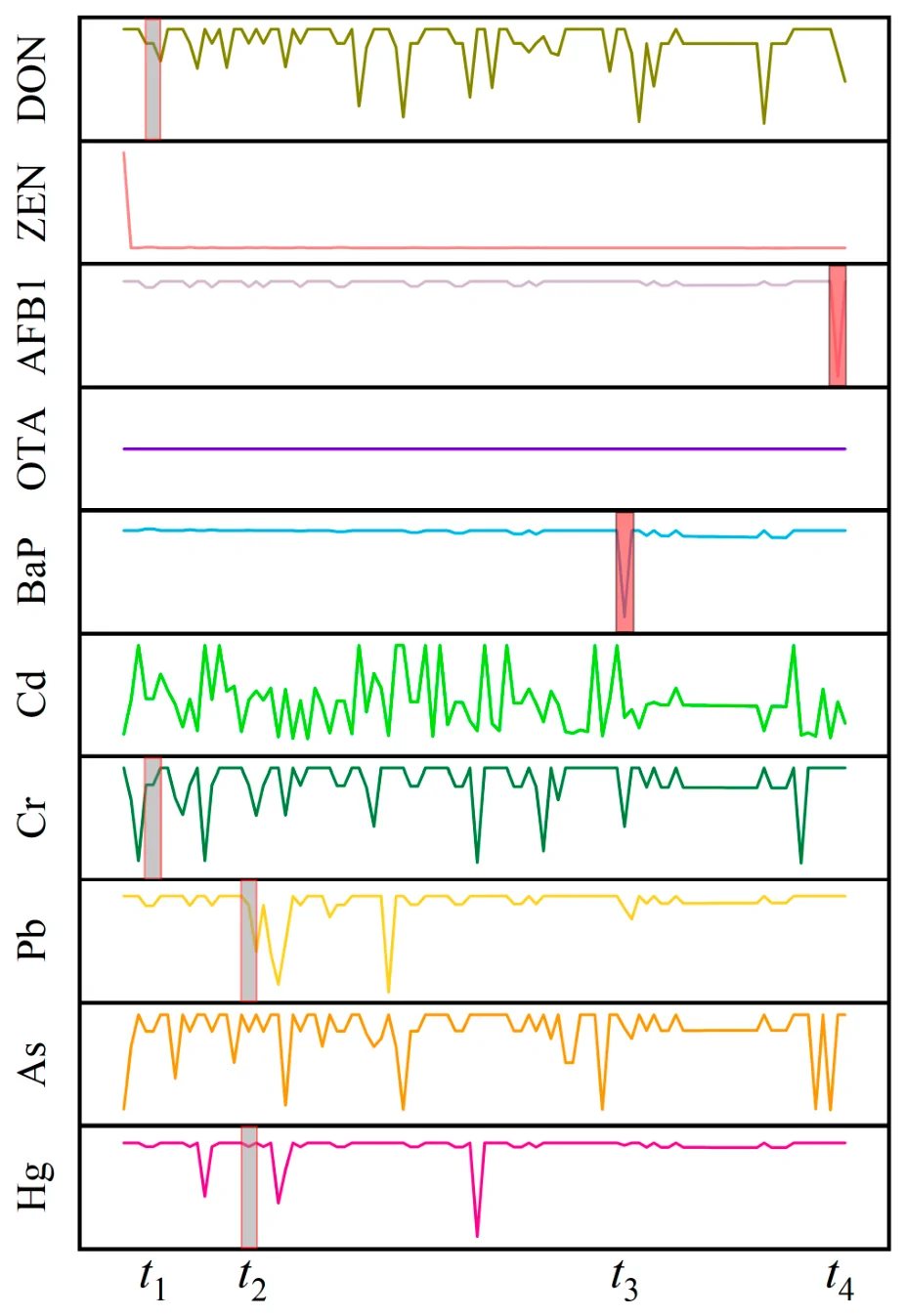
Anomaly detection results for the wheat flour transportation hazard factor dataset. The red shaded areas indicate abnormalities caused by single-variable mutations, while the gray shaded areas indicate abnormalities caused by synergistic changes in multiple variables.

In this section, we investigate the effectiveness of the proposed MSTUAD in anomaly detection through a case study. Figure 7 illustrates selected anomalous time periods from the wheat flour transportation hazard factor dataset. The data samples at time points *t*_1_, *t*_2_, *t*_3_, and *t*_4_ represent anomalies identified by the proposed model and correspond to confirmed abnormal events.

As shown in Figure 7, the trends of AFB1 and BaP exhibit relatively stable patterns with periodic fluctuations, which generally correspond to cyclical variations in transportation batches and environmental conditions in real-world scenarios. When significant changes in AFB1 and BaP occur at time points t3 and t4, both the proposed MSTUAD and existing anomaly detection models are able to identify these anomalies. This is because such anomalies exhibit prominent univariate characteristics with large fluctuation amplitudes, classifying them as easily observable anomalies. Therefore, most anomaly detection models demonstrate strong detection capability for this type of anomaly.

At the same time, the temporal trends of Cr and DON were largely consistent throughout most periods and exhibited a strong correlation, suggesting a potential synergistic coupling relationship. However, at time points t1 and t2, the relative variation patterns of Cr and DON, as well as Pb and Hg, exhibited noticeable deviations, suggesting that the previously learned coupling relationships among hazard factors were disrupted and resulted in anomalous states. Since other anomaly detection models do not explicitly model the complex dependencies among hazard factors, they may fail to capture abrupt changes in coupling relationships and consequently have difficulty detecting such anomalies.

In contrast, the proposed MSTUAD model utilizes graph structure learning to characterize the mutual coupling relationships among hazard factors within each time window, and it employs a multi-scale spatial–temporal graph model to dynamically model and update evolving dependency structures. As a result, when coupling relationships among hazard factors change, MSTUAD can effectively identify potential anomalies induced by these structural shifts—even when individual hazard factors exhibit normal behavior, thereby substantially enhancing anomaly detection accuracy.

## 4. Discussion

1. Model adaptability in special environments

This paper investigates hazard factors during wheat flour transportation. By constructing feature maps to characterize the coupling relationships and designing a multi-scale spatial–temporal graph model to extract spatial–temporal features from these maps, we employ a reconstruction model based on variational autoencoders to learn latent representations of hazard factors’ spatial–temporal characteristics for capturing the intrinsic features of normal wheat flour. However, during actual transportation, wheat flour safety is also influenced by external contextual factors, including transportation environments, geographic conditions, and packaging characteristics, which may interact with hazard factors through complex mechanisms. For example, extreme environmental conditions, such as high-altitude and extremely cold environments, may cause packaging materials to become brittle, deform, or rupture, compromising packaging integrity and increasing the risk of moisture penetration and microbial contamination, which may further promote mold growth and mycotoxin accumulation. Meanwhile, hot and humid climates, such as those in Southeast Asia, may accelerate microbial metabolic activity and mycotoxin production, causing wheat flour with identical initial hazard levels to exhibit different risk profiles. Furthermore, even when the measured hazard levels of samples from different regions are comparable, differences in transportation routes and climatic conditions may result in divergent safety outcomes. Therefore, a key challenge in food safety research is how to incorporate multi-source heterogeneous information to move beyond risk analysis frameworks driven solely by individual hazard factor data and how to effectively integrate structured and unstructured data to uncover the intrinsic relationships among diverse influencing factors.

2. Trade-off between model accuracy and efficiency

As shown in Figure 5, compared with w/o MSTGM, MSTUAD exhibits superior anomaly detection accuracy during wheat flour transportation. This improvement arises because the multi-scale spatial–temporal graph model can effectively extract dynamic variation features of hazard factors across multiple time scales and environmental conditions, enabling multi-granularity perception and information fusion, facilitating more accurate identification of potential anomaly patterns. Meanwhile, multi-scale information is integrated to characterize temperature and humidity variations induced by seasonal and geographical factors, which enhances the robustness and reliability of wheat flour anomaly detection. However, multi-scale spatial–temporal graph models typically integrate parallel and stacked conventional convolutional layers, which inevitably increase the number of model parameters and computational complexity, thereby limiting their applicability in large-scale, real-time, and high-speed risk detection scenarios. Previous studies have demonstrated that depthwise separable convolution, which decomposes standard convolution into depthwise and pointwise operations, can substantially reduce both the number of parameters and computational costs [42]. Therefore, integrating depthwise separable convolution with multi-scale approaches to reduce computational overhead while preserving detection accuracy remains an important research challenge.

3. Adjustment and optimization of key parameters

As discussed in Section 3.1.3, the proposed MSTUAD in this paper incorporates key parameters such as the number of graph neural network layers, the embedding dimensions, and the time window size, which jointly affect the overall performance of wheat flour anomaly detection. Figure 6 illustrates that comprehensive experimental evaluation facilitates the identification of optimal hyperparameter configurations, thereby improving the accuracy of anomaly detection in wheat flour. However, this study primarily relies on theoretical analysis and iterative experimentation, without an automated hyperparameter search mechanism. This dependence leads to a time-consuming and inefficient tuning process that is difficult to adapt to diverse and dynamically evolving scenarios. Furthermore, manual parameter tuning is susceptible to overfitting across different real-world scenarios, thereby undermining the robustness and stability of the model. Deep Q-Network (DQN) has the capacity to autonomously learn optimization strategies within complex, high-dimensional, and uncertain search spaces, thereby enabling dynamic exploration and global optimization of hyperparameter configurations. Therefore, designing an automatic hyperparameter optimization mechanism based on DQN to enhance model tuning efficiency and generalization capability constitutes an important direction for future research.

## 5. Conclusions and Future Work

This paper proposes an MSTUAD framework for wheat flour anomaly detection. It uses a multi-scale convolutional attention to capture both short-term dynamic changes and long-term temporal trend information across multiple time scales. Meanwhile, the multi-scale spatial–temporal graph model and reconstruction representation can effectively capture the spatial–temporal characteristics of wheat flour hazard factors while learning the features of normal samples through reconstruction. Comparative experiments conducted on the wheat flour transportation hazard factor dataset demonstrate that MSTUAD attains an F1-score of 0.8455, surpassing all baseline methods. Furthermore, MSTUAD achieves the highest F1-score on three public benchmark datasets (SMAP: 0.9803, MSL: 0.9629, and SWaT: 0.9520), as well as the best AUC on the SMAP and MSL datasets, further validating its effectiveness and generalization capability in anomaly detection tasks.

Future work will focus on collecting multi-source heterogeneous data and integrating them with wheat flour hazard factor detection data to construct a wheat flour safety risk assessment system based on heterogeneous information fusion. This strategy is expected to improve the capability to address diverse and complex abnormal scenarios encountered during wheat flour transportation and storage.

## Figures and Tables

**Table 1:** The national food safety standard for wheat flour.

Category	Items	Limited Value	National Standard
Heavy metals	Hg	0.02 mg/kg	GB 2762-2025
	As	0.5 mg/kg	GB 2762-2025
	Pb	0.2 mg/kg	GB 2762-2025
	Cd	0.1 mg/kg	GB 2762-2025
	Cr	1.0 mg/kg	GB 2762-2025
Mycotoxins	ZEN	60.0 μg/kg	GB 2761-2017
	DON	1000 μg/kg	GB 2761-2017
	OTA	5.0 μg/kg	GB 2761-2017
	AFB1	5.0 μg/kg	GB 2761-2017
Others	BaP	2.0 μg/kg	GB 2762-2025

**Table 2:** Details of the compared methods.

Competitors	Basis	Publication and Year
IF [27]	Classical	ICDM, 2008
LSTM-NDT [28]	Prediction-based	KDD, 2018
GDN [29]		AAAI, 2021
AGCSL [30]		IEEE TIM, 2024
OmniAnomaly [31]	Reconstruction-based	KDD, 2019
MSCRED [32]		AAAI, 2019
MTAD-GAT [33]		ICDM, 2020
TranAD [34]		VLDB, 2022
CAE-M [35]		IEEE TKDE, 2023
MUTANT [36]		KBS, 2023
MEMTO [37]		NIPS, 2023
DTAAD [38]		KBS, 2024
LFTSAD [39]		TNNLS, 2025
TransDe [40]	Contrastive learning-based	NN, 2025

**Table 3:** The comparative experiments with the state-of-the-art anomaly detection model.

Method	*AUC*	*Pre*	*Rec*	*F1*
IF	0.5231 ± 0.005	0.6657 ± 0.025	0.1207 ± 0.005	0.2044 ± 0.008
LSTM-NDT	0.4916 ± 0.000	0.5468 ± 0.000	0.9056 ± 0.000	0.6819 ± 0.000
GDN	0.5936 ± 0.001	0.6036 ± 0.000	0.9709 ± 0.001	0.7444 ± 0.000
AGCSL	0.6061 ± 0.009	0.6104 ± 0.005	**0.9851 ± 0.004**	0.7537 ± 0.005
OmniAnomaly	0.5937 ± 0.003	0.6067 ± 0.002	0.9230 ± 0.008	0.7321 ± 0.002
MSCRED	0.5646 ± 0.009	0.5870 ± 0.005	0.9583 ± 0.014	0.7279 ± 0.004
MTAD-GAT	0.5647 ± 0.005	0.5861 ± 0.003	0.9791 ± 0.010	0.7333 ± 0.004
TranAD	0.6131 ± 0.026	0.7847 ± 0.016	0.3439 ± 0.087	0.4712 ± 0.082
CAE-M	0.5681 ± 0.010	0.5885 ± 0.006	0.9702 ± 0.008	0.7326 ± 0.006
MUTANT	0.7187 ± 0.014	**0.8227 ± 0.010**	0.7775 ± 0.013	0.7994 ± 0.010
MEMTO	0.7132 ± 0.031	0.7733 ± 0.029	0.6810 ± 0.056	0.7231 ± 0.038
DTAAD	0.5984 ± 0.003	0.6069 ± 0.002	0.9650 ± 0.004	0.7451 ± 0.002
LFTSAD	0.6259 ± 0.008	0.7375 ± 0.004	0.9507 ± 0.003	0.8306 ± 0.004
TransDe	0.5294 ± 0.003	0.6957 ± 0.007	0.6067 ± 0.002	0.6482 ± 0.012
**MSTUAD**	**0.7243 ± 0.011**	0.8037 ± 0.006	0.8896 ± 0.015	**0.8444 ± 0.0** **0** **9**

**Table 4:** Dataset Information.

Dataset	Dimensions	Train	Test	Anomalies (%)
MSL	55 (27)	58,317	73,729	10.72
SMAP	25 (55)	135,183	427,617	13.13
SWaT	51 (1)	99,000	89,984	12.15

**Table 5:** Anomaly detection accuracy in terms of *AUC*, *Pre*, *Rec* and *F1* on three datasets.

Method	MSL				SMAP				SWaT			
	*AUC*	*Pre*	*Rec*	*F1*	*AUC*	*Pre*	*Rec*	*F1*	*AUC*	*Pre*	*Rec*	*F1*
IF	0.5201	0.1452	0.1307	0.1376	0.4865	0.0976	0.0761	0.0855	0.8178	0.7996	0.6583	0.7221
LSTM-NDT	0.6378	0.7585	0.2864	0.4158	0.7412	0.2988	0.7355	0.4249	0.8391	0.7197	0.7168	0.7182
GDN	0.8355	0.7646	0.6962	0.7288	0.7759	0.8489	0.5665	0.6795	0.8619	0.9765	0.7263	0.8330
AGCSL	0.8928	0.7831	0.8121	0.7973	0.7762	0.8548	0.5665	0.6814	0.5705	0.1388	0.9930	0.2435
OmniAnomaly	0.9540	0.7820	0.9388	0.8533	0.7755	0.8500	0.5656	0.6792	0.8708	0.8959	0.7537	0.8187
MSCRED	0.8362	0.7451	0.7006	0.7222	0.7913	0.8363	0.5997	0.6985	0.9026	0.4968	0.9364	0.6492
MTAD-GAT	0.9230	0.7463	0.8813	0.8082	0.7762	0.8542	0.5666	0.6813	0.8446	0.9769	0.6914	0.8097
TranAD	0.8914	0.5605	0.8623	0.6794	0.7822	0.8912	0.5748	0.6988	0.8766	0.5850	0.8351	0.6880
CAE-M	0.6903	0.8404	0.3893	0.5321	0.7782	0.7087	0.5921	0.6452	0.9413	0.6399	0.9570	0.7669
MUTANT	0.9880	0.8847	**0.9968**	0.9374	0.9753	**0.9855**	0.9531	0.9691	0.9594	0.9584	0.9269	0.9424
MEMTO	0.9707	0.9325	0.9495	0.9409	0.9717	0.9493	0.9508	0.9501	0.9430	0.9401	0.8938	0.9164
DTAAD	0.9378	0.5640	0.9634	0.7115	0.7782	0.9192	0.5638	0.6989	0.8796	**0.9869**	0.7606	0.8591
LFTSAD	0.8881	0.9145	0.7854	0.8451	0.9315	0.9432	0.8707	0.9055	0.9400	0.9416	0.8876	0.9138
TransDe	0.9891	0.9242	0.9882	0.9551	0.9899	0.9343	**0.9900**	0.9614	**0.9827**	0.9286	**0.9756**	0.9515
**MSTUAD**	**0.9902**	**0.9359**	0.9914	**0.9629**	**0.9909**	0.9740	0.9866	**0.9803**	0.9717	0.9505	0.9535	**0.9520**

**Table 6:** Comparison of model complexity of different methods.

Method	Params (M)	Train Time (s)	F1 (%)
IF	-	0.17	20.82
LSTM-NDT	0.0204	25.59	68.19
GDN	**0.0015**	260.41	74.38
AGCSL	0.0082	137.41	74.60
OmniAnomaly	0.0149	236.71	73.35
MSCRED	1.2374	302.22	71.08
MTAD-GAT	0.1299	351.31	73.13
TranAD	0.0106	6.13	58.09
CAE-M	0.0072	146.21	71.08
MUTANT	0.0654	434	78.25
MEMTO	5.8169	7.88	67.57
DTAAD	0.0039	6.24	74.65
LFTSAD	0.0069	212.17	83.24
TransDe	0.8271	408.02	64.82
MSTUAD	4.0592	2922	**84.44**

Note: The best results are highlighted in bold, and the second-best results are underlined.

## Data Availability

Data will be made available on request.

## References

[1] Srivastava R., Singh Y., White J.C., Dhankher O.P. (2024). Mitigating toxic metals contamination in foods: Bridging knowledge gaps for addressing food safety. Trends. Food. Sci. Tech..

[2] Melendreras García C., Soldado Cabezuelo A., Ferrero Martín F., Valledor Llopis M., Ortíz-Gómez I., Manuel Costa-Fernández J. (2025). An Affordable IoAT Colorimetric Sensor Based on NanoMOFs for On-Site Food Safety Detection. IEEE Trans. Instrum. Meas..

[3] Song C., Wu Z., Gray J., Meng Z. (2024). An RFID-Powered Multisensing Fusion Industrial IoT System for Food Quality Assessment and Sensing. IEEE Trans. Ind. Inform..

[4] Han Y., Liu J., Pan F., Ni Q., Ma B., Geng Z. (2025). Synthesized minority Oversampling Technique-Reverse k-nearest Neighbors-K-Dimensional Tree for dairy food safety risk evaluation. Expert Syst. Appl..

[5] Oteiza J.M., Prez V.E., Pereyra D., Jaureguiberry M.V., Sánchez G., Sant’Ana A.S., Barril P.A. (2022). Occurrence of Norovirus, Rotavirus, Hepatitis a virus, and Enterovirus in Berries in Argentina. Food Environ. Virol..

[6] He X., Liu X., Wu P., Zhang L., Zhou W., Zhang Q., Zhang J. (2023). Reduction of pathogenic bacteria from irrigation water through a copper-loaded porous ceramic emitter. Environ. Pollut..

[7] Yan J., Sun L., Zuo E., Zhong J., Li T., Chen C., Chen C., Lv X. (2024). An explainable unsupervised risk early warning framework based on the empirical cumulative distribution function: Application to dairy safety. Food Res. Int..

[8] Ma B., Han Y., Cui S., Geng Z., Li H., Chu C. (2020). Risk early warning and control of food safety based on an improved analytic hierarchy process integrating quality control analysis method. Food Control.

[9] Han Y., Cui S., Geng Z., Chu C., Chen K., Wang Y. (2019). Food quality and safety risk assessment using a novel HMM method based on GRA. Food Control.

[10] Wang X., Bouzembrak Y., Lansink A.O., van der Fels-Klerx H.J. (2022). Application of machine learning to the monitoring and prediction of food safety: A review. Compr. Rev. Food Sci. Food Saf..

[11] Sheng W., Jiang H., Yang Z., Zhao L., Jin J. (2025). A safety risk assessment method based on conditionally constrained game theory and adaptive ensemble learning: Application to wheat flour and rice. Food Res. Int..

[12] Geng Z., Shang D., Han Y., Zhong Y. (2019). Early warning modeling and analysis based on a deep radial basis function neural network integrating an analytic hierarchy process: A case study for food safety. Food Control.

[13] Geng Z., Wang X., Jiang Y., Han Y., Ma B., Chu C. (2023). Novel IAPSO-LSTM neural network for risk analysis and early warning of food safety. Expert Syst. Appl..

[14] Zuo E., Du X., Aysa A., Lv X., Muhammat M., Zhao Y., Ubul K. (2022). Anomaly Score-Based Risk Early Warning System for Rapidly Controlling Food Safety Risk. Foods.

[15] Zuo E., Yan J., Aysa A., Chen C., Chen C., Ma H., Lv X., Ubul K. (2023). SUCOLA: Self-adaptive structure refinement unsupervised contrastive learning framework for food safety risk early warning. Eng. Appl. Artif. Intell..

[16] China Administration for Market Regulation. https://www.samr.gov.cn.

[17] (2025). National Food Safety Standards—Limits of Contaminants in Food.

[18] (2017). National Food Safety Standards—Limits of Mycotoxins in Food.

[19] Kinney J.B., Atwal G.S. (2014). Equitability, mutual information, and the maximal information coefficient. Proc. Natl. Acad. Sci. USA.

[20] Guo M.-H., Lu C.-Z., Hou Q., Liu Z., Cheng M.-M., Hu S.-M. (2022). SegNeXt: Rethinking Convolutional Attention Design for Semantic Segmentation. Adv. Neural Inf. Process. Syst..

[21] Li G., Müller M., Qian G., Delgadillo I.C., Abualshour A., Thabet A., Ghanem B. (2023). DeepGCNs: Making GCNs Go as Deep as CNNs. IEEE Trans. Pattern Anal. Mach. Intell..

[22] Beck M., Pöppel K., Spanring M., Auer A., Prudnikova O., Kopp M.K., Klambauer G., Brandstetter J., Hochreiter S. (2024). xLSTM: Extended Long Short-Term Memory. The Thirty-Eighth Annual Conference on Neural Information Processing Systems, Vancouver, BC, Canada, 10–15 December 2024.

[23] Kingma D.P., Welling M. (2022). Auto-Encoding Variational Bayes. arXiv.

[24] Liu Z., Wang Y., Vaidya S., Ruehle F., Halverson J., Soljacic M., Hou T.Y., Tegmark M. KAN: Kolmogorov-Arnold Networks. Proceedings of the Thirteenth International Conference on Learning Representations.

[25] Kingma D.P., Welling M. (2014). Stochastic Gradient VB and the Variational Auto-Encoder. arXiv.

[26] Kim S., Choi K., Choi H.-S., Lee B., Yoon S. (2022). Towards a Rigorous Evaluation of Time-Series Anomaly Detection. Proc. AAAI Conf. Artif. Intell..

[27] Liu F.T., Ting K.M., Zhou Z.-H. Isolation Forest. Proceedings of the Eighth IEEE International Conference on Data Mining.

[28] Hundman K., Constantinou V., Laporte C., Colwell I., Soderstrom T. Detecting Spacecraft Anomalies Using LSTMs and Nonparametric Dynamic Thresholding. Proceedings of the 24th ACM SIGKDD International Conference on Knowledge Discovery & Data Mining.

[29] Deng A., Hooi B. (2021). Graph Neural Network-Based Anomaly Detection in Multivariate Time Series. Proc. AAAI Conf. Artif. Intell..

[30] Pang H., Wei S., Li Y., Liu T., Zhang H., Qin Y., Zhao Y. (2024). Asymptotic Consistent Graph Structure Learning for Multivariate Time-Series Anomaly Detection. IEEE Trans. Instrum. Meas..

[31] Su Y., Zhao Y., Niu C., Liu R., Sun W., Pei D. Robust Anomaly Detection for Multivariate Time Series through Stochastic Recurrent Neural Network. Proceedings of the 25th ACM SIGKDD International Conference on Knowledge Discovery & Data Mining.

[32] Zhang C., Song D., Chen Y., Feng X., Lumezanu C., Cheng W., Ni J., Zong B., Chen H., Chawla N.V. A deep neural network for unsupervised anomaly detection and diagnosis in multivariate time series data. Proceedings of the Thirty-Third AAAI Conference on Artificial Intelligence.

[33] Zhao H., Wang Y., Duan J., Huang C., Cao D., Tong Y., Xu B., Bai J., Tong J., Zhang Q. Multivariate Time-Series Anomaly Detection via Graph Attention Network. Proceedings of the IEEE International Conference on Data Mining (ICDM).

[34] Tuli S., Casale G., Jennings N.R. TranAD: Deep transformer networks for anomaly detection in multivariate time series data. Proceedings of the VLDB Endowment.

[35] Zhang Y., Chen Y., Wang J., Pan Z. (2023). Unsupervised Deep Anomaly Detection for Multi-Sensor Time-Series Signals. IEEE Trans. Knowl. Data Eng..

[36] Shi Y., Wang B., Yu Y., Tang X., Huang C., Dong J. (2023). Robust anomaly detection for multivariate time series through temporal GCNs and attention-based VAE. Knowl.-Based Syst..

[37] Song J., Kim K., Oh J., Cho S. MEMTO: Memory-guided transformer for multivariate time series anomaly detection. Proceedings of the 37th International Conference on Neural Information Processing Systems.

[38] Yu L.-R., Lu Q.-H., Xue Y. (2024). DTAAD: Dual Tcn-attention networks for anomaly detection in multivariate time series data. Knowl.-Based Syst..

[39] Chen L., Tang J., Zou Y., Liu X., Xie X., Deng G. (2025). Lightweight and Fast Time-Series Anomaly Detection via Point-Level and Sequence-Level Reconstruction Discrepancy. IEEE Trans. Neural Netw. Learn. Syst..

[40] Zhang W., Luo C. (2025). Decomposition-based multi-scale transformer framework for time series anomaly detection. Neural Netw..

[41] Mathur A.P., Tippenhauer N.O. (2016). SWaT: A water treatment testbed for research and training on ICS security. International Workshop on Cyber-Physical Systems for Smart Water Networks.

[42] Sandler M., Howard A., Zhu M., Zhmoginov A., Chen L.-C. MobileNetV2: Inverted Residuals and Linear Bottlenecks. Proceedings of the IEEE/CVF Conference on Computer Vision and Pattern Recognition.

